# Metabolomic changes in severe acute malnutrition suggest hepatic oxidative stress: a secondary analysis

**DOI:** 10.1016/j.nutres.2021.05.005

**Published:** 2021-07

**Authors:** Mariana Parenti, Shannon McClorry, Elizabeth A. Maga, Carolyn M. Slupsky

**Affiliations:** a Department of Nutrition, University of California, Davis, USA; b Department of Animal Science, University of California, Davis, USA; c Department of Food Science and Technology, University of California, Davis, USA

**Keywords:** Malnutrition, Children, Liver, Oxidative stress, Metabolomics, Piglet

## Abstract

Severe acute malnutrition (SAM), due to poor energy and/or protein intake, is associated with poor growth, depressed immune function, and long-term impacts on metabolic function. As the liver is a major metabolic organ and malnutrition poses metabolic stress, we hypothesize that SAM will be associated with alterations in the hepatic metabolome reflective of oxidative stress, gluconeogenesis, and ketogenesis. Thus, the purpose of this secondary analysis was to understand how SAM alters hepatic metabolism using a piglet model.

Weanling piglets were feed either a reference (REF) or protein-energy deficient diet (MAL) for 5 weeks. After dietary treatment MAL piglets were severely underweight (weight-for-age Z-score of -3.29, Welch's *t* test, *P* = .0007), moderately wasted (weight-for-length Z-score of-2.49, Welch's *t* test, *P* = .003), and tended toward higher hepatic triglyceride content (Welch's *t* test, *P* = .07). Hematologic and blood biochemical measurements were assessed at baseline and after dietary treatment. The hepatic metabolome was investigated using ^1^H-NMR spectroscopy. Hepatic concentrations of betaine, cysteine, and glutathione tended to be lower in MAL (Welch's *t* test with FDR correction, *P* < .1), while inosine, lactate, and methionine sulfoxide concentrations were higher in MAL (inosine: *P* = .0448, lactate: *P* = .0258, methionine sulfoxide: *P* = .0337). These changes suggest that SAM is associated with elevated hepatic oxidative stress, increased gluconeogenesis, and alterations in 1-carbon metabolism.

## 1 Introduction

Globally, undernutrition is either directly or indirectly associated with approximately 45% of deaths in children under 5 years of age [1]. Severe acute malnutrition (SAM), which is the result of inadequate dietary intake of protein and/or calories, likely manifests along a clinical spectrum; however, SAM is predominantly classified as severe wasting (weight-for-length Z-score; WLZ >3 Z-scores below the median), edematous malnutrition, or a combination [2,3]. Childhood SAM impairs innate and adaptive immunity [2] and has long-term negative impacts on cognitive development, academic performance, and productivity, and has additionally been associated with metabolic syndrome, diabetes, and cardiovascular risk factors in middle age [4], [5], [6], [7], [8]-9].

In addition to the effects of suboptimal energy and nutrient intake, oxidative stress as a result of SAM has been suggested to further contribute to these long-term developmental impacts [10], [11]-12]. Children with SAM present with depleted erythrocyte glutathione (GSH) concentrations, reduced rates of GSH synthesis, and depressed GSH peroxidase (GPx) activity. Additionally, reduced concentrations of circulating antioxidants, including zinc, selenium, copper, and vitamins A, C, and E are observed in children with SAM [13], [14], [15]-16]. Malnourishment is further compounded by the deleterious effects of SAM on the intestine, leading to flattened villi and impaired nutrient absorption, as well as increased intestinal permeability [17]. SAM frequently co-occurs with megaloblastic or microcytic anemia, suggesting the additional burden of one or more micronutrient deficiencies [18].

Piglets have been recently been recognized as a more powerful translational model than rodents in human nutritional and gastrointestinal research because they have similar digestive anatomy and physiology, as well as hepatic function [19], [20], [21]-22]. Carbohydrates, amino acids, and lipids are digested and metabolized similarly in pigs and humans and the digestion products have similar effects on gastrointestinal development [21]. Several piglet models of SAM have been developed over the last decade [23], [24], [25], [26]-27]. Garas et al [23] developed, and subsequently replicated [24], a model of SAM rooted in protein-energy malnutrition, in which severely malnourished piglets exhibited several clinical signs of SAM observed in humans, including alterations in intestinal morphology and function, hematology, and serum biochemistry. Animals in this model consistently present with severe wasting; significant intestinal changes including decreased crypt depth, decreased thickness of the lamina propria, increased transcellular and paracellular permeability, and decreased weight and length of small intestine [23,24]. One notable discrepancy was that the malnourished piglets in this model did not exhibit hypoalbuminemia [23,24], which is typically observed in children with SAM [13].

Impaired hepatic function and hepatic steatosis have been observed in malnourished children for many decades [28], [29]-30], and a tendency toward hepatic fat accumulation was observed in another piglet model of SAM [25]. Hypoalbuminemia, a well-known sign of malnutrition, is suggestive of reduced liver synthetic function [13]. Furthermore, SAM alters hepatic metabolism in human children, resulting in prolonged drug half-lives or decreased clearance of pharmacological agents [31] and impaired bile synthesis [32]. Studies in rodent models of dietary protein deficiency have corroborated these observations, including steatohepatitis tied to mitochondrial and peroxisomal dysfunction in rats [33] and decreased biliary function in mice [34]. In both models, abnormalities in liver metabolism were accompanied by increased markers of oxidative stress [33,34].

Currently, there are limited studies of diet-induced SAM using a swine model, and to our knowledge, no studies have investigated the impact of SAM on the hepatic metabolome during the post-weaning period. Previous reports using this model have shown significant alterations in the gastrointestinal tract of malnourished piglets with SAM [23,24], including alterations in morphology and increased permeability, which could alter the influx of digestive and microbial byproducts into hepatic portal circulation. Furthermore, malnutrition poses a physiological stress requiring alterations in metabolic pathways to support energy metabolism. Therefore, we hypothesize that SAM will be associated with alterations in hepatic metabolism, including markers of oxidative stress, ketogenesis, and gluconeogenesis. As such, the purpose of this study is to further describe the swine model previously reported on by Garas, et al. [23,24] by characterizing the hepatic metabolome in SAM using ^1^H-NMR metabolomics and determining differences in hepatic triglyceride concentrations.

## 2 Methods and materials

### 2.1 Animals and experimental diets

This study is a secondary analysis of samples from 2 previously reported studies that sought to understand the effects of transgenic milk supplementation in malnutrition [23,24]. The work presented here examines the effect of SAM on hepatic metabolism by comparing only the unsupplemented malnourished controls with the adequately fed piglets from these 2 studies. Briefly, for each study, weanling male Yorkshire-Hampshire cross piglets were supplied by the UC Davis Swine Facility. Shortly after birth, all piglets received an injection of 100 mg of elemental iron in the form of an iron dextran, which is common practice in the swine industry for the prevention of iron-deficiency anemia during the pre-weaning period. All piglets nursed for 21 days prior to being weaned onto a weaning diet, which is high in zinc, for 4 days. At this point (study day 0, referred to as baseline), 3.5-week-old weanling piglets were weighed and whole blood was collected for a complete blood count. Litter- and weight-matched piglets were then randomized into the adequately fed reference (REF) or unsupplemented malnourished (MAL) diet groups. From baseline, REF piglets (*n* = 6 per study) were fed a commercial diet containing 18.7% protein and 6% fat *ad libitum*. REF piglets were individually housed in the first study, whereas they remained in group housing in the second. MAL piglets (*n* = 6 per study) were fed a diet containing 14.2% protein and 6% fat and restricted to a daily feed intake equivalent to 3% of their body weight, which is approximately half of the required feed intake. MAL piglets were fed twice daily (morning and evening), with equal portions at each meal. For both studies, MAL piglets were individually housed in order to control dietary intake. The ingredients and nutrient content of the weaning, REF, and MAL diets can be found in Tables 1 and 2, respectively. As a result of restricted feed intake, the MAL diet did not provide sufficient quantities of energy, protein, measured amino acids, and some minerals to meet needs [35]. Piglets were provided with ad libitum access to water. Growth was monitored throughout the studies using snout-to-rump length measurements recorded at baseline, and on study days 21 and 35. Prior to necropsy on study day 35 (8.5 weeks of age), animals were fasted overnight, anesthetized with tiletamine HCl and zolazepam HCl (Telazol; Zoetis Inc.) prior to whole blood and serum collection, and then euthanized using sodium pentobarbital (Fatal Plus; Vortech Pharmaceuticals Ltd.). Liver was collected immediately and snap-frozen. Liver and serum samples were stored at -80 °C until analyses. The care and use of all animals in this study followed the Association for Assessment and Accreditation of Laboratory Animal Care (AAALAC) approved conditions and were approved by the UC Davis Institutional Animal Care and Use Committee.

**Table 1:** Ingredient composition of experimental diets

Ingredient, g/kg	REF	MAL
Corn	582.5	630.0
Soybean meal	250.0	161.9
Distillers dried grains	50.0	0
Fat	30.0	33.0
Limestone	11.5	13.5
Phosphate	8.5	23.3
L-Lysine	4.0	0.5
Salt	4.0	5.0
L-Threonine	0.7	0
Wheat millrun	50.0	130.3
Lincomycin	2.5	0
Vit-mineral premix	2.5	1.5
DL-Methionine	1.1	0
Potassium Chloride	1.0	0
Zinc oxide	1.0	0
Copper sulfate	0.7	0
Choline chloride	0	1.0
*Total*	1000.0	1000.0

During the experimental period, reference (REF) piglets were fed the REF diet ad libitum, whereas malnourished (MAL) piglets were fed the MAL diet in restricted amounts of 3% of body weight daily.

**Table 2:** Nutrient composition of the weaning, malnourished, and reference diets, as well as nutritional requirements for a 9 kg piglet

Nutrient	Weaning Diet (per kg)	REF Diet (per kg)	MAL Diet (per kg)	Daily MAL diet for 9 kg piglet	Min daily requirement of 9 kg piglet
Metabolizable energy (kcal)	3305	3342	3181	859*	1592
Protein (g)	206.9	187.3	144.1	-	-
*Calculated Nitrogen (g)*	*33.1*	*30*	*23.0*	6.22*	*15.4*
Arginine (g)	nm	nm	9.3	2.5*	3.2
Lysine (g)	14.9	12.8	7.8	2.1*	7.2
Methionine (g)	nm	nm	2.8	0.8*	2.1
Threonine (g)	nm	nm	6.1	1.7*	4.4
Fat (g)	54.5	59.1	61.9	-	-
Calcium (g)	8.00	7.00	10.25	2.77*	3.75
Copper (mg)	173.0	174.8	24.07	6.50	2.81
Iodine (mg)	nm	nm	0.60	0.16	0.07
Iron (mg)	nm	nm	137.4	37.1*	46.8
Magnesium (g)	nm	nm	1.67	0.45	0.19
Manganese (mg)	nm	nm	70.37	19.0	1.87
Phosphorus (g)	6.4	5.8	9.02	2.44*	3.04
Potassium (g)	nm	nm	6.31	1.70	1.31
Selenium (mg)	nm	nm	0.45	0.12*	0.14
Zinc (mg)	4500	852.1	151.0	40.8*	46.8
Biotin (mg)	nm	nm	0.36	0.10	0.02
Vitamin A (IU)	nm	nm	12812	3459	1030
Vitamin D (IU)	nm	nm	1766	477	103
Vitamin E (IU)	66.0	66.0	82.0	22.1	7.5

Abbreviation: nm, not measured.

All piglets were weaned on to the weaning diet, which they received ad libitum for 4 days prior to randomization. The nutrient composition of both the reference (REF) and malnourished (MAL) feeds are provided. The REF diet was formulated to meet the needs of growing piglets and was fed ad libitum, however, the malnourished (MAL) diet was fed under restriction, such that each piglet received a weight of feed equal to 3% of their body weight. The average weight of MAL piglets at the end of the study was approximately 9 kg, thus requirements of a 9 kg piglet and the nutrient composition of 270 g of MAL feed have been provided for comparison. An asterisk (*) indicates nutrients in MAL diet that did not meet the daily needs of starting piglets per *Nutrition Requirements of Swine, Eleventh Revised Edition*[35].

### 2.2 Hematological analysis

Whole blood obtained on study days 0 and 35 was used to analyze hematology and blood chemistry panels by the UC Davis Veterinary Medicine Teaching Hospital Clinical Diagnostic Laboratory as previously described [24].

### 2.3 Liver polar metabolite extraction

Liver samples were received frozen and stored at -80°C until metabolite extraction. Approximately 250 mg of each sample was mixed with 10 mM phosphate buffer in a 6:1 volume:weight ratio. Samples were homogenized using a FastPrep-24 (MP Biomedicals, Santa Ana, CA, USA), vortexed, and centrifuged (Centrifuge 5415 R, Eppendorf, Hamburg, Germany). The supernatant was collected and further centrifuged before being filtered through an Amicon Ultra-0.5 mL 3,000 MW centrifugal filter (Millipore, Burlington, MA, USA) that had been washed 3 times with ultra-pure water to remove glycerol. Of the resulting filtrate, 207 µL was aliquoted and 23 µL of internal standard (Chenomx, Edmonton, AB), containing 5.0 mM 3-(trimethylsilyl)-1-propanesulfonic acid-d_6_, (DSS-d_6_), 0.2% NaN_3_ (to inhibit microbial growth) and 99.8% D_2_O (to serve as an NMR lock), was added. The samples were stored overnight at 4°C. Each sample's pH was adjusted to 6.8 ± 0.1, and 180 µL was loaded into a 3 mm NMR tube (Bruker, Billerica, MA). Samples were stored at 4°C until spectral acquisition (within the same day).

### 2.4 Serum polar metabolite extraction

Serum samples were stored at -80°C until extraction. After thawing on ice, serum was filtered through Amicon centrifugal filters prepared as described above, and samples were prepared as described in section 2.3.

### 2.5 ^1^H-NMR spectral acquisition

^1^H-NMR spectra at 25°C were collected using a Bruker Avance 600 MHz spectrometer (Bruker, Billerica, MA, USA) using the noesypr1d pulse sequence with parameters as previously described [36]. Spectra were manually phase- and baseline-corrected using Chenomx NMR Processor (v. 8.1, Chenomx, Edmonton, AB, USA). Metabolite concentrations for each sample type (liver or serum) were quantified by a single researcher using Chenomx Profiler (v. 8.1, Chenomx, Edmonton, AB, USA). The quantification relies on the internal standard (DSS-d_6_) to determine each metabolite's concentration using the Chenomx 600 MHz Library (v. 10), which allows for both absolute quantification and the ability to quantify many compounds within the spectrum [37].

### 2.6 Hepatic triglyceride assay

Approximately 100 mg of liver tissue was finely minced then homogenized in 1 mL of IGEPAL CO-630 (Sigma Aldrich, St. Louis, MO, USA) using a FastPrep-24 (MP Biomedicals, Santa Ana, CA, USA). Hepatic triglyceride content was determined using a colometric triglyceride quantification kit (Sigma Aldrich, St. Louis, MO, USA) according to manufacturer instruction using a Synergy H1 Microplate reader (BioTek, Winooski, VT, USA).

### 2.7 Statistical analyses

As this was a secondary analysis, all piglets from the original studies with remaining liver tissue or serum samples were analyzed. For liver metabolomics, this resulted in sample sizes of *n* = 5 (REF; 3 individually-housed, 2 group-housed) and *n* = 6 (MAL), and for serum metabolomics sample sizes were *n* = 2 (REF; both group-housed) and *n* = 6 (MAL). In the analysis of liver triglyceride concentration, sample sizes were *n* = 5 (REF) and *n* = 5 (MAL), as the metabolomics analyses consumed all available tissue from 1 MAL piglet. In our analysis of the liver metabolome, this sample size provides the sensitivity to detect Hedges’ *g* effect sizes of at least 1.75 with α = 0.05 and 80% power.

Descriptive statistics were calculated for piglet weight, hematology, and blood biochemistry on study days 0 and 35. Weight-for-age Z-scores (WAZ), length-for-age Z-scores (LAZ), and weight-for-length Z-scores (WLZ) were calculated by subtracting the mean value of the REF group from each piglet's measured and dividing by the measure's standard deviation in the REF group. Welch's *t* tests were used to assess differences between dietary treatment groups for anthropometry, hematology, and blood biochemistry at each time point, as well as hepatic triglyceride content. The resulting *p*-values are reported with *P*< .05 considered significant.

Hepatic and serum metabolite concentrations are expressed as nmol/g tissue weight and µmol/L, respectively. Serum and hepatic metabolite concentrations were log_10_-transformed and principal component analysis (PCA) was conducted on each tissue type separately using the *prcomp* function in the R *stats* package. Welch's *t.test* was conducted using the *t.test* function in *stats* package in order to analyze the effects of malnutrition on untransformed metabolite concentrations. The *p-*values resulting from the *t* tests were corrected for false discovery rate (FDR) based on the number of quantified metabolites using the Benjamini–Hochberg procedure. Due to the small sample sizes, uncorrected *p*-values are reported in addition to FDR-corrected *p*-values (denoted as *Fp*) for metabolite concentrations, and statistical significance was defined as *Fp* < 0.05, while a 0.05 < *Fp* < 0.10 represents a statistical trend. Effect sizes (*ES*) were determined using Hedge's *g* method in the R package *effsizes*. Based on the absolute value of the effect size, strength was classified as follows: 0.50 to 0.8, moderate; 0.8 to 1.2, strong; and ≥1.20, very strong. All statistical analyses were performed using R (v 3.5.1) and RStudio (v 1.1.453) and visualizations were produced using the *ggplot2* package.

## 3 Results

### 3.1 Piglet characteristics, hematology, and blood biochemistry

Piglet anthropometric measurements, hematology, and blood biochemistry for the 11 piglets with available liver samples are shown in Table 3. On study day 0, anthropometric measurements did not statistically differ between MAL (*n* = 6) and REF (*n* = 5) piglets. Additionally, with the exception of a trend toward higher phosphorus concentrations in the MAL group (*P*= .06), no differences in blood counts or biochemistry measurements were noted between the 2 groups. After 35 days on their respective diets, MAL piglets weighed approximately half as much as REF piglets (WAZ = -3.29, *P* = .0007), tended to have shorter snout-to-rump length (LAZ = -1.22, *P*= .09), and had significantly lower weight-to-length ratio (WLZ = -2.49, *P*= .003). Hematological measurements indicated MAL piglets had decreased MCH (*P*= .04) and increased RBC count (*P*= .009) relative to REF piglets. Additionally, several differences were noted in the blood biochemistry of MAL and REF piglets at the week 5 timepoint. In comparison to REF piglets, MAL piglets had lower phosphorus (*P*= .03), calcium (*P*= .01), and glucose (*P*= .009) concentrations, as well as higher albumin (*P*= .05). Total hepatic triglyceride content was 9.87 ± 2.15 µmol/g in MAL piglets and 7.51 ± 0.53 µmol/g in REF piglets (*P*= .07).

**Table 3:** Physical characteristics, blood counts, and blood biochemistry of piglets at baseline and after 5 weeks on study diets

		Study Day 0			Study Day 35		
	Expected Range	REF (*n* = 5)	MAL (*n* = 6)	*P* value	REF (*n* = 5)	MAL (*n* = 6)	*P* value
Weight (kg)	–	6.1 ± 1.0	6.1 ± 0.8	.9291	19.7 ± 3.2	9.1 ± 1.6	.0007
WAZ	–	0.00 ± 1.00	-0.05 ± 0.80	.9291	0.00 ± 1.00	-3.29 ± 0.51	.0007
Length (cm)	–	53.6 ± 2.6	54.4 ± 3.5	.6657	69.5 ± 11.9	55.1 ± 13.6	.0930
LAZ	–	0.00 ± 1.00	0.31 ± 1.32	.6657	0.00 ± 1.00	-1.22 ± 1.15	.0930
Weight: Length	–	0.113 ± 0.015	0.111 ± 0.012	.7852	0.288 ± 0.048	0.168 ± 0.026	.0026
WLZ	–	0.00 ± 1.00	-0.157 ± 0.81	.7852	0.00 ± 1.00	-2.49 ± 0.55	.0026
*Hematology analytes*							
Hgb (g/dL)	8.8-12.7	10.2 ± 1.3	10.2 ± 1.0	.9672	12.2 ± 0.9	12.8 ± 0.9	.2644
Hct (%)	28.3-42.7	34.3 ± 3.4	34.9 ± 2.6	.7341	39.0 ± 3.6	40.6 ± 2.8	.4397
MCV (fL)	38.4-59.3	51.4 ± 4.2	51.3 ± 2.6	.9787	51.6 ± 6.0	48.7 ± 2.3	.3612
MCH (pg)	11.1-18.4	15.2 ± 1.7	14.9 ± 1.0	.6887	16.7 ± 1.0	15.4 ± 0.5	.0351
MCHC (g/dL)	27.9-32.4	29.6 ± 1.2	29.0 ± 0.8	.3504	31.3 ± 1.0	31.7 ± 0.7	.5494
RBC (10^6^/μL)	5.52-9.11	6.67 ± 0.21	6.81 ± 0.32	.4113	7.25 ± 0.55	8.31 ± 0.43	.0093
WBC (10^3^/µL)	5.44-25.19	8.32 ± 3.03	6.95 ± 3.03	.4726	21.27 ± 4.33	12.41 ± 2.21	.0067
Neutrophils (10^3^/µL)	0.81-13.40	3.63 ± 2.99	3.15 ± 2.41	.7800	7.07 ± 1.05	3.55 ± 2.08	.0072
Lymphocytes (10^3^/µL)	3.81-14.92	4.16 ± 1.52	3.16 ± 0.78	.2336	12.55 ± 3.01	8.73 ± 1.83	.0451
Monocytes (/µL)	219-1705	230 ± 109	172 ± 149	.3178	1005 ± 571	391 ± 112	.0731
Eosinophils (/µL)	45-481	230 ± 50	259 ± 115	.5919	533 ± 329	303 ± 205	.2191
Basophils (/µL)	14-146	14.2 ± 9.3	12.3 ± 6.2	.7123	66.2 ± 25.1	55.2 ± 27.2	.5033
Platelets (10^5^/µL)	2.09-8.73	5.65 ± 1.94	6.15 ± 1.77	.6692	6.99 ± 2.57	4.83 ± 1.94	.1636
*Biochemical analytes*							
Anion Gap (mM)	14-29	22.2 ± 4.8	24.2 ± 2.5	.4377	26.4 ± 4.6	22.5 ± 3.0	.1508
Sodium (mM)	131-151	139 ± 2.3	140 ± 3.1	.7950	141 ± 2.2	139 ± 8.7	.5856
Potassium (mM)	3.7-6.1	4.36 ± 0.47	4.53 ± 0.64	.6198	5.12 ± 1.29	5.37 ± 0.58	.7086
Chloride (mM)	93-108	99.8 ± 2.2	98.7 ± 1.4	.3471	99.0 ± 1.0	96.7 ± 9.0	.5546
Bicarbonate (mM)	19-31	21.6 ± 5.1	21.5 ± 2.7	.9698	20.6 ± 3.9	25.0 ± 2.2	.0661
Phosphorus (mg/dL)	6.3-11.5	7.46 ± 0.54	8.28 ± 0.72	.0588	10.02 ± 0.98	8.32 ± 1.20	.0292
Calcium (mg/dL)	9.9-12.5	11.1 ± 0.3	10.9 ± 0.5	.5325	11.7 ± 0.3	10.9 ± 0.5	.0148
BUN (mg/dL)	4-18	9.40 ± 1.95	11.50 ± 3.73	.2664	6.00 ± 2.55	9.17 ± 4.83	.2030
Creatinine (mg/dL)	0.5-1.1	1.14 ± 0.23	1.20 ± 0.13	.6212	0.82 ± 0.15	1.02 ± 0.18	.0809
BUN:Creatinine	–	8.77 ± 3.44	9.59 ± 3.00	.6906	7.24 ± 2.72	9.40 ± 6.10	.4607
Glucose (mg/dL)	75-136	119 ± 23	116 ± 19	.8360	128 ± 13	98 ± 17	.0094
Total Protein (g/dL)	4.0-5.8	4.74 ± 0.50	4.92 ± 0.44	.5553	5.38 ± 0.13	5.57 ± 0.39	.3094
Albumin (g/dL)	3.1-4.8	3.80 ± 0.39	3.80 ± 0.56	1.0000	3.78 ± 0.28	4.40 ± 0.56	.0466
Globulin (g/dL)	0.3-1.7	0.94 ± 0.34	1.12 ± 0.49	.4972	1.60 ± 0.29	1.17 ± 0.37	.0570
AST (U/L)	13-111	44.4 ± 17.2	89.0 ± 72.1	.1956	90.4 ± 58.5	47.8 ± 24.2	.1869
Creatine kinase (U/L)	153-5427	1053 ± 699	3595 ± 4029	.1856	6513 ± 4872	1618 ± 1416	.0872
Alkaline phosphatase (U/L)	130-513	455 ± 87	447 ± 100	.8853	314 ± 74	251 ± 53	.1510
GGT (U/L)	33-94	55.4 ± 15.5	62.2 ± 6.8	.4037	85.8 ± 57.6	51.7 ± 22.3	.2669
SDH (U/L)	0-1.7	0.80 ±1.10	0.17 ± 0.41	.2763	0.00 ± 0.00	0.67 ± 0.52	.0250

Abbreviations: AST, aspartate aminotransferase; BUN, blood urea nitrogen; GGT, γ-glutamyltransferase; Hct, hematocrit; Hgb, hemoglobin; LAZ, length-for-age Z-score; MCH, mean corpuscular hemoglobin; MCHC, mean corpuscular hemoglobin concentration; MCV, mean corpuscular volume; RBC, red blood cells; SDH, sorbitol dehydrogenase; WAZ, weight-for-age Z-score; WLZ, weight-for-length Z-score; WBC, white blood cells.

At 3.5 weeks of age (Study Day 0), weanling piglets were randomized to the reference (REF) or malnourished (MAL) diets. Weight, hematological parameters, and blood chemistry are reported as means ± standard deviation and differences between the 2 groups at each time point were assessed using Welch's *t* test. Expected values were extracted from [35].

### 3.2 Metabolomics analyses

**Fig. 1:**
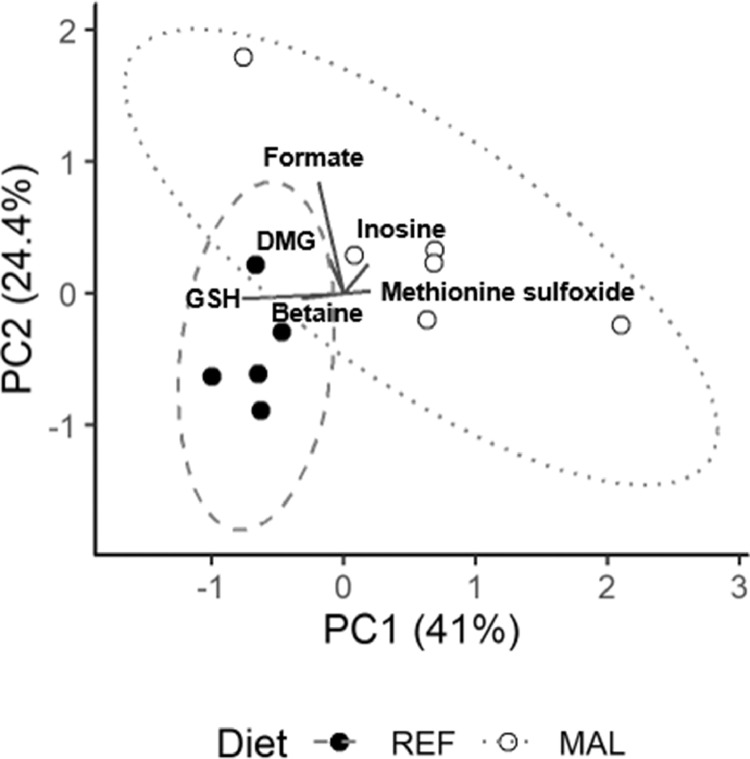
Malnutrition influences the hepatic metabolome. Principal components analysis (PCA) was performed on the log_10_-transformed hepatic metabolome. A biplot with 95% confidence ellipses indicates separation between reference piglets (REF, filled circles, *n* = 5) and malnourished (MAL, open circles, *n* = 6) piglets along PC1. The labelled metabolites are those with the greatest influence on PC1 and PC2. DMG, dimethylglycine; GSH, glutathione.

A biplot from PCA of the hepatic metabolome indicates differences between REF and MAL piglets (Fig. 1). Hepatic metabolites exhibiting significant differences between the REF and MAL groups are shown in Table 4. Additionally, data for all measured hepatic (Supplemental Table S1) and serum metabolites (Supplemental Table S2) are included in the online supplementary materials. All analyses were also completed on only those piglets from the first study, where both groups were individually housed, to assess whether observed differences were the result of different housing conditions of the REF group between the 2 studies. These analyses reflected similar patterns to those presented here with piglets from both studies (data not shown).

**Table 4:** Hepatic metabolites which are significantly or tend to be altered in SAM

Metabolite	REF (nmol/g)	MAL (nmol/g)	*P*-value	*Fp*-value	ES
3-Hydroxybutyrate	21.9 ± 5.5	35.0 ± 9.3	.0200	0.0660	1.52
Betaine	737.2 ± 268.7	315.3 ± 278.4	.0317	0.0888	1.41
Cysteine	1844.9 ± 412.3	1010.3 ± 526.3	.0160	0.0610	1.60
Glutathione	351.7 ± 166.8	89.9 ± 82.3	.0204	0.0660	**1.89**
Guanosine	84.4 ± 35.5	169.3 ± 41.1	.0053	**0.0417**	**2.00**
Hippurate	28.9 ± 5.5	58.9 ± 8.5	.0001	**0.0034**	**3.71**
Inosine	602.4 ± 377.5	1366.4 ± 384.2	.0096	**0.0448**	**1.83**
Lactate	8009.5 ± 1125.9	4373.3 ± 363.4	.0012	**0.0258**	**4.18**
Mannose	1487.5 ± 324.9	2009.1 ± 322.9	.0278	0.0834	1.46
Methionine sulfoxide	8.9 ± 3.9	22.2 ± 6.6	.0032	**0.0337**	**2.17**
Methionine sulfoxide: Methionine	0.0147 ± 0.0058	0.0328 ± 0.0085	.0025	**0.0337**	**2.24**
*myo*-Inositol	1180.1 ± 201.2	748.7 ± 263.9	.0131	0.0549	1.66
Niacinamide	762.9 ± 101.8	963.8 ± 78.5	.0080	**0.0422**	**2.07**
Ornithine	1140.1 ± 79.6	1567.5 ± 253.7	.0080	**0.0422**	**1.97**
Serine	2988.4 ± 741.5	5574.6 ± 1461.4	.0060	**0.0417**	**1.97**

Metabolites that significantly (*Fp* < 0.05) or tend (*Fp* < 0.1) to differ in the hepatic metabolome of MAL piglets. Concentrations are expressed as means ± standard deviation for each group, reference (REF, *n* = 5) and malnourished (MAL, *n* = 6). Welch's *t* test was used to compare the REF and MAL groups. The resulting *P* values were reported with *P* values corrected for False Discovery Rate (*Fp*, calculated using 42 metabolites). Hedges' *g* is reported as a measure of effect size (*ES*). Bolded text indicates *Fp* < 0.05 or Hedges’ *g* > 1.75, which is the threshold for sensitivity in these analyses.

#### 3.2.1 Glutathione synthesis and 1-Carbon Metabolism

**Fig. 2:**
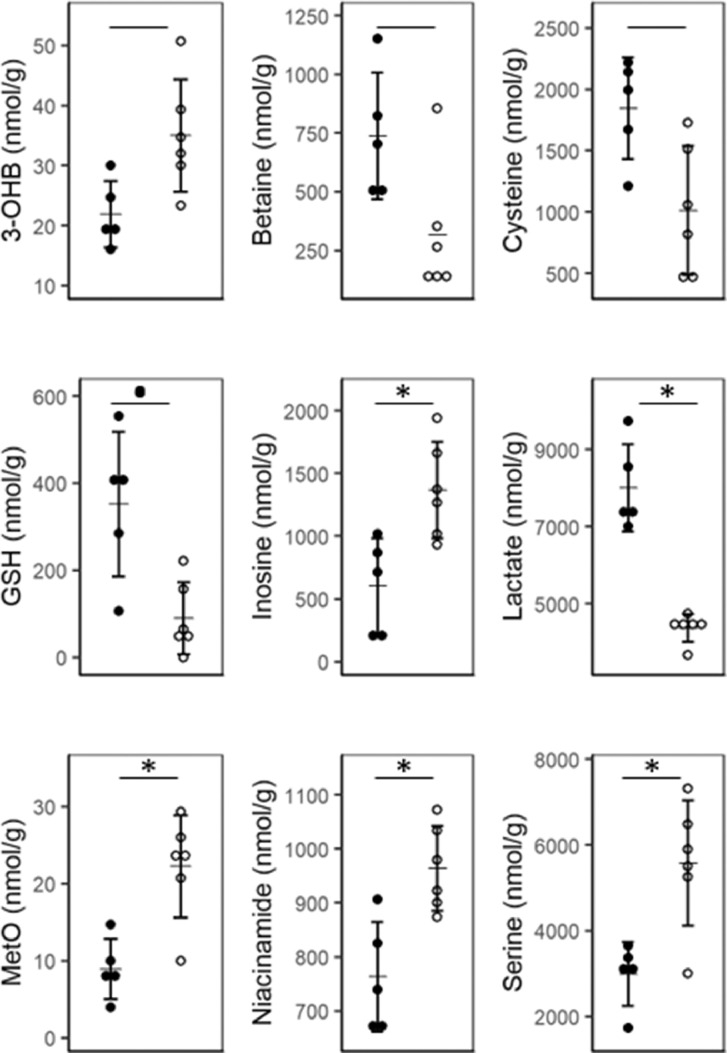
Hepatic metabolites that differ in malnourishment. Dot plots (means ± SD) of the hepatic metabolites that significantly or tended to differ between the reference (REF, filled circles, *n* = 5) and malnourished (MAL, open circles, *n* = 6) piglets (Welch's *t* test with FDR-correction; * indicates *Fp* < 0.05 and ● indicates *Fp* < 0.1). 3-OHB, 3-hydroxybutyrate; GSH, glutathione, MetO, methionine sulfoxide.

**Fig. 3:**
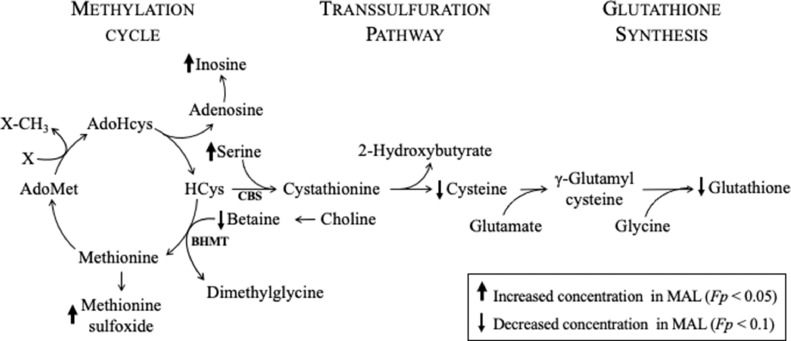
1-Carbon metabolism (1CM) and glutathione (GSH) synthesis. Methylation is connected to the folate cycle through the remethylation of homocysteine (HCys) to methionine via hepatic betaine-homocysteine methyltransferase (BHMT) using betaine. Methionine can be used to make S-adenosylmethionine (AdoMet), the universal methyl donor. After being used in the methylation reaction, it becomes S-adenosylhomocysteine (AdoHCys), which in turn becomes adenosine and homocysteine. Homocysteine can either be remethylated to methionine or enter the transsulfuration cycle to create cystathionine via cystathionine β synthase (CBS), and ultimately glutathione. Arrows indicate if the metabolite tended to be elevated (up) or depressed (down) in the MAL liver (FDR-corrected Welch's *t* test; *Fp* < 0.1).

In the liver, the re-methylation of homocysteine to methionine relies on the conversion of betaine to dimethylglycine by betaine-homocysteine methyltransferase (BHMT) [38]. Betaine concentrations tended to be depressed in the MAL group relative to REF group, with very strong effect sizes (Fig. 2, Table 4, *P*= .03, *Fp* = 0.09, *ES* = 1.41). However, hepatic choline, methionine, and dimethylglycine did not significantly differ between MAL and REF piglets (Supplemental Table S1). The methylation cycle of 1-carbon metabolism (1CM) and GSH synthesis are connected through the transsulfuration pathway (Fig. 3). Hepatic serine concentrations were higher in MAL compared to REF, with a strong effect size (Fig. 2, Table 4, *P*= .006, *Fp* = 0.04, *ES* = 1.97). In contrast, hepatic cysteine tended to be lower in the MAL compared to REF piglets (Fig. 2, Table 4, *P*= .02, *Fp* = 0.06, *ES* = 1.60). No differences in hepatic formate, glutamate, or glycine concentrations were observed between groups (Supplemental Table S1).

#### 3.2.2 Oxidative stress

Hepatic GSH concentrations in the REF group were nearly 4 times greater than in the MAL group (Fig. 2, Table 4, *P*= .02, *Fp* = 0.07, *ES* = 1.89). MAL piglets also exhibited hepatic methionine sulfoxide concentrations double those of the REF piglets (Fig. 2, Table 4, *P* = .003, *Fp* = 0.03, *ES* = 2.17) and a significantly greater hepatic methionine sulfoxide-to-methionine concentration ratio (Table 4, *P* = .002, *Fp* = 0.03, *ES* = 2.24). Hepatic inosine concentrations were doubled in MAL piglets relative to REF piglets, with very strong effect sizes (Fig. 2, Table 4, *P*= . 01, *Fp* = 0.04, *ES* = 1.83). Hepatic guanosine and inosine concentrations were doubled in the MAL piglets relative to REF piglets (Table 4; guanosine: *P*= .005, *FP* = 0.04, *ES* = 2.00; inosine: *P*= .01, *FP* = 0.04, *ES* = 1.83).

#### 3.2.3 Energy metabolism

Hepatic lactate concentrations were significantly lower in MAL piglets (Fig. 2, Table 4, *P*= .001, *Fp* = 0.03, *ES* = 4.18); however, no differences were noted in the citric acid cycle intermediates succinate, fumarate, and malate (Supplemental Table S1). Niacinamide, a component of nicotinamide adenine dinucleotide (NAD), was elevated in MAL relative to REF piglets (Fig. 2, Table 4, *P*= .008, *FP* = 0.04, *ES* = 2.07). The ketone 3-hydroxybutyrate tended to be elevated in the MAL relative to REF piglets with a strong effect size (Table 4, *P*= .02, *Fp* = 0.07, *ES* = 1.62).

## 4 Discussion

### 4.1 Piglet model of severe acute malnutrition

Clinically, the 2 main manifestations of SAM (formerly referred to as protein-energy malnutrition) are severe wasting or edematous malnutrition. Severe wasting, which has also been referred to as marasmus, is defined as a weight-for-height Z-score (WLZ) 3 standard deviations below the median. On the other hand, edematous malnutrition, or Kwashiorkor, is characterized by pitting edema, hypoalbuminemia, and hepatic steatosis [3]. The piglet model of malnutrition presented in this secondary analysis has been previously validated, with malnourished piglets exhibiting moderate to severe wasting, as well as the expected alterations in hematology, serum biochemistry, and intestinal morphology and function [23,24]. In this analysis, piglets on the MAL diet were moderately wasted (mean WLZ = -2.49) and severely underweight (mean WAZ = -3.29). There was also a tendency toward stunting after 5 weeks on the MAL diet, suggesting that prolonged exposure to the MAL diet would result in chronic malnutrition.

Lowered immune parameters are frequently observed in malnutrition [39], with MAL piglets in this study exhibiting significantly lower WBC, neutrophil, and lymphocyte counts, as well as a trend toward decreased monocytes. MAL piglets in this model were noted to have significantly greater albumin concentrations than REF piglets, whereas hypoalbuminemia is often observed in children with SAM [3]. A recent meta-analysis of malnourished, but otherwise healthy adults indicated that albumin levels generally remain within normal range, thus albumin is not indicative of nutritional status [40]. Instead, the hallmark hypoalbuminemia observed in children with SAM may be the result of the increased frequency and severity of infection [41], as albumin is a negative acute phase protein. While MAL piglets in this study exhibited elevated albumin levels, it should be noted that values for both MAL and REF piglets remained within normal ranges [42].

Since changes in immune parameters in MAL piglets were not due to infection, the observed changes in the hepatic metabolome presented are likely due to SAM. Furthermore, there was no evidence of hepatic injury in MAL piglets after 5 weeks on a protein-energy deficient diet. While elevated sorbitol dehydrogenase (SDH) is potentially indicative of acute liver injury [43], the observed values remain within the expected range. Additionally, no differences were noted in blood aspartate aminotransferase (AST) or alkaline phosphatase between the 2 treatment groups.

Despite a limited sample number for REF piglets in the serum metabolome analyses, the changes noted in the serum metabolome (Supplemental Table S2) of MAL piglets align with those of previous studies in piglets [26] and malnourished children [44,45]. In another porcine model of SAM, plasma concentrations of glucose, glutamate, and methionine were significantly lower in malnourished piglets after 5 weeks of a protein-restricted diet [26]. Additionally, low serum methionine and glutamate concentrations have been reported in malnourished children [44,45], as has low fasting blood glucose. Thus, the purpose of this secondary analysis was to investigate the alterations in hepatic metabolism associated with this model of SAM.

### 4.2 Alterations in 1CM and transsulfuration lead to depressed GSH

Alterations in the hepatic metabolome suggest elevated oxidative stress in malnourished piglets. Notably, hepatic GSH concentration in the malnourished group was less than one-fifth of the concentration of the reference group. GSH synthesis and 1CM are connected through the transsulfuration pathway (Fig. 3).

Despite insufficient dietary methionine in the MAL diet, hepatic methionine concentrations did not differ between the REF and MAL groups. This might be due to increased activity of betaine-homocysteine methyltransferase (BHMT), and decreased activity of cystathionine β-synthase (CBS) to maintain hepatic methionine levels. Previous work in an avian model showed that a diet deficient in methionine resulted in increased BHMT activity and decreased cystathionine β-synthase (CBS) activity [46]. In our study, we observed lower hepatic betaine concentrations in MAL piglets. As part of 1CM, betaine is used as a methyl donor by BHMT to remethylate homocysteine to methionine [38] suggesting increased BHMT activity. We also observed that MAL piglets exhibited significantly higher hepatic serine concentrations, as well as lower cysteine and GSH concentrations, suggesting that less homocysteine entered the transsulfuration pathway, and further that CBS activity was decreased. Cysteine has previously been recognized as the limiting amino acid in GSH synthesis [47]. A previous study showed that supplementation with cysteine increases both erythrocyte GSH concentration and synthesis in children with SAM [48].

RBC synthesis is highly dependent on the folate/methionine cycles, thus the elevated RBC counts observed in MAL piglets was unexpected. However, it has been shown that rats fed a protein-deficient diet supplemented with vitamin B_6_ have elevated RBC compared to rats fed either a protein-sufficient diet or an unsupplemented protein-deficient diet [49]. Unfortunately, the vitamin B_6_ content of each diet was not measured.

### 4.3 Oxidative stress in malnutrition

GSH is an important antioxidant involved in detoxification and maintenance of oxidative status [47]. Hepatic GSH was observed to be more than 70% lower in MAL piglets relative to healthy REF piglets, which is consistent with the observations of previous work in rat models [50,51]. While we were unable to quantify GSH in serum, studies in children [14,15], rats [52], and piglets [27] indicate that circulating GSH concentrations are also depressed in SAM. GSH is a substrate for a variety of enzymes, including glutathione peroxidase (GPx), which catalyzes the reduction of hydroperoxides while oxidizing GSH to its disulfide form. Since the activity of GPx is depressed in protein-energy malnutrition [14] and iron deficiency [53], and GPx contains selenium [47], which was inadequate in the MAL diet, the lower hepatic concentration suggests an increased oxidative load and/or decreased synthesis. The purines guanosine and inosine also have antioxidant properties [54], [55]-56]. Both metabolites reach hepatic concentrations in MAL piglets almost double those of REF piglets, which may represent a response to elevated oxidative stress. Furthermore, the elevated hepatic concentration of methionine sulfoxide and ratio of methionine sulfoxide-to-methionine each indicate an increased oxidative load in MAL piglets [57], suggesting a failure of antioxidants like GSH, guanosine, and inosine to maintain normal cellular function. Indeed, the MAL diet provided inadequate zinc to meet the needs of growing piglets, a mineral essential in the function of copper-zinc superoxide dismutase, which catalyzes the conversion of superoxide to hydrogen peroxide [58]. Thus, both inadequate amino acids and micronutrients may have contributed to elevated oxidative stress observed in the MAL piglets in this study.

Fatty infiltration of the liver has been reported in human and animal models of childhood SAM [25,[28], [29]-30,59,60] and hepatic GSH depletion has been shown to induce steatohepatitis in rodent models [61]. Furthermore, oxidative stress is a key underlying factor in the development of non-alcoholic fatty liver disease [62]. While hepatic triglyceride concentrations only tended to be elevated in this model of malnutrition, metabolic perturbations may point to fatty infiltration of the liver. Diets low in *myo*-inositol have been linked to increased liver triglycerides in rodent models and supplementation has been associated with further reducing liver triglycerides, as reviewed by Pani et al. [63]. A trend in piglets on the MAL diet toward lower concentrations of hepatic *myo*-inositol, which can also be endogenously produced from glucose, suggests alterations in hepatic lipid metabolism. While *myo*-inositol content in the diet was not measured, the energy restriction in this piglet model of malnutrition may restrict endogenous production of *myo*-inositol. Additionally, niacin supplementation has been proposed as a potential therapeutic or preventative measure for non-alcoholic fatty liver disease [64]. In human liver biopsies, nicotinic acid was inversely associated with the severity of steatohepatitis and supplementation with nicotinic acid reduced steatohepatitis in a mouse model of non-alcoholic fatty liver disease [65]. Thus, elevated hepatic concentrations of niacinamide, a form of niacin, in MAL piglets may also suggest metabolic adaption to fatty infiltration of the liver.

Since the liver is the site of essential systemic metabolic functions, hepatic oxidative stress may lead to detrimental systemic effects. In cholestatic liver disease, oxidative stress is not confined to the liver, and evidence of lipid peroxidation is found in diverse tissues, including the heart and brain [66]. Furthermore, protein malnutrition in rodents not only leads to neuroanatomical and neurochemical changes [12], but elevated lipid peroxidation and lower total antioxidant capacity also leads to oxidative damage in the brain [67,68]. Oxidative stress is also a risk factor of cardiometabolic disturbances [10]. Thus, early-life oxidative damage may be a source of later life cognitive deficits and metabolic perturbations associated with childhood SAM.

### 4.4 Alterations in energy metabolism in malnutrition

In malnutrition, the liver must compensate for inadequate energy and nutrient intake. In this study, MAL piglets were noted to have lower blood glucose concentrations, though hepatic concentrations of glucose and citric acid cycle intermediates did not differ between the REF and MAL piglets. Thus, another source of nutrients must be utilized to maintain energy production, which was likely body stores of protein and fat via gluconeogenesis and ketogenesis, respectively. In this study, hepatic ornithine concentrations in MAL piglets were significantly greater than in REF piglets. Additionally, MAL piglets were observed to have hepatic urea concentrations approximately 1.7 times greater than REF piglets, and while these differences did not achieve statistical significance, they did exhibit strong effect sizes. Taken together, these changes suggest increased flux through the urea cycle, which may be in response to the use of glucogenic amino acids as an energy substrate. Although few differences in hepatic amino acid concentrations were observed, malnourished piglets in the initial validation trial had significantly elevated BUN, creatinine, and BUN-to-creatinine ratios [23]; however, MAL piglets in this secondary analysis only trended toward elevated creatinine concentrations, which may be due to the limited sample size. Moderate dehydration has been shown to decrease functional hepatic nitrogen clearance in healthy men [69]. Nonetheless, while these differences may suggest alterations in hydration status, both BUN and creatinine levels are within normal ranges [42]. Thus, the combination of elevated creatinine and indications of increased flux through the urea cycle suggest catabolism of skeletal muscle to meet needs.

Inosine, in addition to its antioxidant effects, has also been shown to stimulate glycogenolysis, gluconeogenesis, and ureagenesis in the liver [70]. In this study, mean hepatic inosine concentrations were double in MAL piglets, and in keeping with previous findings, hepatic lactate was nearly halved, suggesting increased use as a substrate for gluconeogenesis. Furthermore, the concentration of 3-hydroxybutyrate tended to be elevated in the malnourished liver which suggests an increase in ketogenesis and is consistent with elevated gluconeogenesis [71]. These results indicate shifts in energy production and metabolism in malnutrition. Interestingly, lower endogenous glucose production has been noted in children with edematous, but not non-edematous, malnutrition [72].

### 4.5 Limitations and future directions

A few limitations exist regarding the interpretation of the results presented in this study. First, as this study was a secondary analysis, we were only able to include piglets for which samples remained, which resulted in lower sample sizes particularly for analysis of the serum metabolome for the REF piglets. The dietary composition of the MAL diet also provided challenges with respect to interpretation since when given in restricted quantities, it was both protein and energy deficient, and also provided inadequate amounts of the minerals iron, calcium, phosphorus, selenium, and zinc (at ~80% of the daily requirement), as well as some essential amino acids (methionine, lysine, and threonine at ~35% of the daily requirement and arginine at ~78% of the daily requirement). Despite the MAL diet providing only 80% of the required iron content, none of the piglets exhibited hemoglobin concentrations indicative of borderline (≤ 8 g/dL) or overt (≤7 g/dL) anemia [31]. This is likely due to the fact that prior to the start of the MAL diet, the piglets had sufficient iron stores to carry them through the 5-week period. Similarly, since calcium, phosphorus, and selenium are micronutrients that can also be stored in the body, the slightly lower micronutrient content of the diet (~80% of the daily requirement) may have been enough to prevent overt deficiency. Inadequate dietary calcium and phosphorous were reflected in lower serum concentrations of these minerals in MAL piglets, though concentrations remained within normal ranges [42]. The observed trend toward higher bicarbonate in MAL piglets could represent a method of compensation for the lower phosphorus to maintain acid-base balance in the blood. Furthermore, the mild dietary inadequacy of selenium in the MAL diet may have been spared as a result of high dietary vitamin E [73]. Although copper levels in the MAL diet were lower than those of the REF diet, copper needs were still met in the MAL diet despite restricted feed intake. The micronutrient of concern in this study was zinc, which was at 87% of the daily requirement, but since zinc is not stored in the body, we cannot rule out that a 13% deficiency over 5 weeks would not impact metabolism and impair growth [74]. Furthermore, the concentrations of many of the water-soluble vitamins in the diet were not provided by the manufacturer. As such, potential deficiencies in folate, vitamin B_6_, or vitamin B_12_ could contribute to the observed alterations in hepatic metabolism in MAL. However, we believe that the observations reported here are likely due to the overt deficiency in several essential amino acids and protein which were at ~1/3 of the daily requirement. Nonetheless, micronutrient deficiencies are often present concurrently with SAM in children [18], and thus this model is relevant to the issues observed in regions with SAM.

Malnutrition is well-known to be associated with alterations in the host microbiome [75]. Hippurate is formed through the conjugation of benzoate and glycine in both the liver and kidneys [76], with benzoate originating from microbial metabolism of dietary components or direct ingestion as a dietary preservative [77]. Studies of malnutrition have noted alterations in circulating or urinary hippurate [26,^78^,79]. As such, we speculate that the higher hepatic hippurate concentrations observed in MAL piglets compared to REF may be the result of dietary differences or alterations in the gut microbiome, though further investigation is warranted.

As piglets are social animals, the potential effect of individually housing the majority of the piglets is worth noting. It has been shown that acute social isolation induces stress in piglets and has been associated with increased cortisol [80,81]. Cortisol is known to induce gluconeogenesis, and chronic stress is associated with markers of oxidative stress in humans [82], both of which could contribute to outcomes observed in this study. However, as only 2 of the REF piglets were group housed, with the remaining REF and all MAL piglets being individually housed, individual housing is unlikely to be the sole contribution to the observed differences between MAL and REF piglets.

Using ^1^H-NMR spectrometry, we used targeted metabolomic techniques to characterize systemic and hepatic metabolism in a piglet model of malnutrition. While the serum metabolome in SAM has previously been investigated, this is the first study to our knowledge elucidating the effects of SAM on the hepatic metabolome in a piglet model. As hypothesized, alterations in the hepatic metabolome of malnourished piglets suggest increases in hepatic oxidative stress, ketogenesis, and gluconeogenesis. Alterations in 1CM were further noted, though future work will be required to disentangle the effects of protein- and energy-restriction from those of potential micronutrient inadequacies and how these changes may be connected to long-term consequences, including hepatic injury, cognitive impairment, and metabolic disturbances. Additionally, piglets provide a valuable translational model for human immune and cognitive development [83,84]. Therefore, this model could be further used to provide insights into the mechanisms, possible treatments, and long-term impacts of childhood SAM.

## Authors’ contributions

Mariana Parenti: Investigation, Formal Analysis, Visualization, Writing (original draft). Shannon M^c^Clorry: Investigation, Formal Analysis, Writing. Elizabeth A. Maga: Conceptualization, Resources, Funding Acquisition. Carolyn M. Slupsky: Conceptualization, Resources, Writing – Review & Editing, Supervision, Funding Acquisition.

## Acknowledgment

The authors would like to thank Lydia Garas for sample generation and provision. Samples used in this work were collected during a study funded by the Bill & Melinda Gates Foundation through the Grand Challenges Explorations Initiative (grant number OPP1067869, awarded to EAM). The Bruker Avance 600 MHz NMR is supported by the National Institutes of Health (grant number RR011973]. This work was also supported by the USDA National Institute of Food and Agriculture Hatch Project 1021411 and from the Kinsella Endowed Chair in Food, Nutrition, and Health (to CMS), and an NIEHS-funded predoctoral fellowship to MP (T32 ES007059). The contents are solely the responsibility of the authors and do not necessarily represent the official views of the NIEHS, NIH, or USDA. Declarations of interest: None.

## Appendix Supplementary materials

Image, application 1
