# Supplementary material for: Metabolomic changes in severe acute malnutrition suggest hepatic oxidative stress: a secondary analysis
Source: Nutr Res. 2021 Jul;91:44–56. doi: 10.1016/j.nutres.2021.05.005 (PMC8311294; doi:10.1016/j.nutres.2021.05.005)
Supplement: Supplementary file 1 [file mmc1.pdf]

Metabolomic changes in severe acute malnutrition suggest hepatic oxidative stress: a secondary analysis

Mariana Parenti[a], Shannon McClorry[a], Elizabeth A. Maga[b], Carolyn M. Slupsky[a,c]

[a]Department of Nutrition,
[b]Department of Animal Science,
[c]Department of Food Science and Technology, University of California, Davis.

Supplemental Table S1. Hepatic metabolites with non-significant differences between REF and MAL groups.

Metabolite	***REF (nmol/g)***	***MAL (nmol/g)***	***p***	***Fp***	***ES***
Acetate	1756.9 ± 1057.4	2198.0 ± 360.0	0.4194	0.5505	0.53
Acetoacetate	22.0 ± 11.8	30.0 ± 12.6	0.3091	0.4808	0.59
Acetone	31.7 ± 8.5	28.5 ± 6.4	0.4890	0.6223	0.42
Alanine	4738.9 ± 911.8	4869.1 ± 684.8	0.8100	0.8723	0.14
Ascorbate	641.1 ± 145.7	730.6 ± 317.4	0.5605	0.6924	0.32
Aspartate	2696.9 ± 409.5	3153.1 ± 484.2	0.1284	0.2568	0.91
Choline	1279.0 ± 386.7	1084.8 ± 84.3	0.3244	0.4867	0.68
Dimethylglycine	9.9 ± 4.7	12.1 ± 8.0	0.5815	0.6978	0.30
Formate	52.6 ± 44.3	677.2 ± 1533.5	0.3644	0.5102	0.50
Fumarate	292.5 ± 41.4	285.4 ± 24.0	0.7286	0.8053	0.21
Glucose	49928.3 ± 3738.3	50293.6 ± 7163.6	0.9326	0.9553	0.05
Glutamate	6375.1 ± 1001.7	8271.7 ± 1564.4	0.0397	0.1041	1.29
Glutamine	2036.5 ± 651.4	1478.1 ± 1000.6	0.2950	0.4808	0.59
Glycine	8084.4 ± 580.1	7946.6 ± 408.5	0.6373	0.7435	0.28
Histidine	782.5 ± 186.3	984.2 ± 142.2	0.0872	0.1928	1.12
Isoleucine	781.3 ± 157.7	883.6 ± 145.1	0.3041	0.4808	0.61
Leucine	1387.9 ± 277.5	1580.6 ± 228.2	0.2559	0.4478	0.69
Malate	1411.6 ± 86.5	1340.8 ± 86.6	0.1961	0.3581	0.78
Methionine	613.3 ± 90.0	670.7 ± 102.7	0.3572	0.5102	0.53
O-Phosphocholine	2252.7 ± 527.3	1994.4 ± 445.6	0.4084	0.5505	0.49

Pantothenate	38.9 ± 8.5	76.4 ± 39.0	0.0664	0.1550	1.15
sn-Glycero-3-phosphocholine	4316.9 ± 1446.3	4283.7 ± 3671.9	0.9812	0.9812	0.01
Succinate	28.7 ± 11.1	28.0 ± 10.7	0.9193	0.9553	0.06
Taurine	2730.8 ± 1256.6	3014.1 ± 1262.2	0.7215	0.8053	0.20
Threonine	1442.5 ± 201.1	1819.0 ± 333.9	0.0514	0.1270	1.20
Urea	1411.3 ± 429.8	2354.2 ± 1168.6	0.1136	0.2385	0.94
Valine	1316.8 ± 243.4	1549.6 ± 258.0	0.1632	0.3116	0.84

For each group, mean concentrations ± standard deviation are given. The groups are reference (REF, n = 5) and malnourished (MAL, n = 6). Welch's T test was used to compare the REF and MAL groups. The resulting *p*-values were reported with *p*-values corrected for False Discovery Rate (*Fp*). Hedges' *g* is reported as a measure of effect size (*ES*).

Supplemental Table S2. Effect of malnutrition on the serum metabolome.

**Metabolite**	**REF (µmol/L)**	**MAL (µmol/L)**	***p*-value**	***Fp***	***ES***
2-Hydroxybutyrate	9.9 ± 0.3	6.7 ± 3.3	0.0649	0.2758	0.92
2-Oxoglutarate	9.7 ± 2.9	6.8 ± 3.4	0.3529	0.6496	0.77
3-Hydroxybutyrate	6.4 ± 2.9	7.0 ± 3.2	0.8262	0.8778	0.17
Acetate	205.1 ± 95.1	157.1 ± 63.9	0.6027	0.7807	0.60
Acetone	2.1 ± 2.9	3.4 ± 1.7	0.6200	0.7807	0.62
Alanine	844.1 ± 233.8	697.8 ± 301.1	0.5433	0.7799	0.44
Arginine	218.4 ± 4.1	226.8 ± 78.8	0.8054	0.8778	0.10
Aspartate	94.3 ± 7.6	69.7 ± 26.6	0.0888	0.3019	0.88
Betaine	190.3 ± 27.1	102.1 ± 48.5	0.0400	0.1943	1.68
Choline	78.3 ± 10.7	49.0 ± 12.2	0.0843	0.3019	2.14
Citrulline	50.6 ± 16.5	117.9 ± 31.7	0.0197	0.1488	1.97
Dimethylglycine	2.6 ± 0.3	2.6 ± 0.4	0.8124	0.8778	0.16
Formate	33.6 ± 7.3	73.2 ± 61.4	0.1788	0.5065	0.61
Fumarate	2.0 ± 0.2	1.6 ± 0.7	0.2108	0.5512	0.59
Glucose	5879.0 ± 329.5	4383.6 ± 766.1	0.0134	0.1488	1.83
Glutamate	577.0 ± 34.4	314.0 ± 86.0	0.0015	0.0526	2.87
Glutamine	332.5 ± 102.2	391.6 ± 116.1	0.5654	0.7799	0.45
Glycine	1068.7 ± 160.8	909.4 ± 264.9	0.3821	0.6496	0.55
Hippurate	11.2 ± 0.9	5.8 ± 4.2	0.0263	0.1488	1.22
Histidine	54.4 ± 2.9	60.1 ± 10.7	0.2846	0.5886	0.50
Inosine	1.1 ± 0.6	1.0 ± 0.9	0.8161	0.8778	0.15
Isoleucine	130.2 ± 21.0	108.1 ± 40.4	0.3776	0.6496	0.51
Lactate	10138.8 ± 1358.7	9017.9 ± 2898.4	0.4999	0.7726	0.36
Leucine	217.7 ± 2.9	155.2 ± 49.1	0.0262	0.1488	1.21
Mannose	73.2 ± 13.4	35.4 ± 9.4	0.1164	0.3598	3.23
Methionine	78.7 ± 10.1	28.7 ± 12.9	0.0234	0.1488	3.50
myo-Inositol	119.7 ± 6.2	110.0 ± 38.4	0.5735	0.7799	0.24
Ornithine	192.5 ± 83.7	193.7 ± 70.6	0.9879	0.9879	0.01

Serine	200.5 ± 7.3	231.1 ± 63.3	0.2943	0.5886	0.46
Succinate	16.3 ± 4.5	10.7 ± 3.5	0.2931	0.5886	1.33
Taurine	271.8 ± 87.4	243.2 ± 96.8	0.7356	0.8778	0.26
Threonine	248.7 ± 101.4	262.0 ± 117.3	0.8915	0.9185	0.10
Urea	1283.7 ± 952.6	2660.5 ± 1446.3	0.2283	0.5544	0.87
Valine	300.9 ± 64.1	251.0 ± 74.3	0.4564	0.7390	0.60

Serum metabolite concentrations are reported as means ± the standard deviation is reported for the reference group (REF, n = 2) and the malnourished group (MAL, n = 6), along with *p*-values, false discovery rate corrected p-values (*Fp*), and Hedges' *g* effect sizes (*ES*).
